# Serum lipids profiling perturbances in patients with ischemic heart disease and ischemic cardiomyopathy

**DOI:** 10.1186/s12944-020-01269-9

**Published:** 2020-05-09

**Authors:** Lin Yang, Liang Wang, Yangyang Deng, Lizhe Sun, Bowen Lou, Zuyi Yuan, Yue Wu, Bo Zhou, Junhui Liu, Jianqing She

**Affiliations:** 1 Vascular surgery Department, First Affiliated Hospital of Xi’an Jiaotong University, Xi’an, China; 2 Department of cardiovascular surgery, The general hospital of Ningxia Medical Univetsity, Yinchuan, China; 3 Cardiovascular Department, First Affiliated Hospital of Medical College, Xi’an Jiaotong University, Xi’an, 710048 People’s Republic of China; 4 Respiratory Department, First Affiliated Hospital of Medical College, Xi’an Jiaotong University, Xi’an, 710048 People’s Republic of China; 5 Diagnostic Department, First Affiliated Hospital of Medical College, Xi’an Jiaotong University, Xi’an, 710048 People’s Republic of China

**Keywords:** Cardiovascular disorders, Ischemic heart disease, Ischemic cardiomyopathy, Lipidomics, Biomarkers

## Abstract

**Background:**

Ischemic heart disease (IHD) is a common cardiovascular disorder associated with inadequate blood supply to the myocardium. Chronic coronary ischemia leads to ischemic cardiomyopathy (ICM). Despite their rising prevalence and morbidity, few studies have discussed the lipids alterations in these patients.

**Methods:**

In this cross-sectional study, we analyzed serum lipids profile in IHD and ICM patients using a lipidomics approach. Consecutive consenting patients admitted to the hospital for IHD and ICM were enrolled. Serum samples were obtained after overnight fasting. Non-targeted metabolomics was applied to demonstrate lipids metabolic profile in control, IHD and ICM patients.

**Results:**

A total of 63 and 62 lipids were detected in negative and positive ion mode respectively. Among them, 16:0 Lyso PI, 18:1 Lyso PI in negative ion mode, and 19:0 Lyso PC, 12:0 SM d18:1/12:0, 15:0 Lyso PC, 17:0 PC, 18:1–18:0 PC in positive ion mode were significantly altered both in IHD and ICM as compared to control. 13:0 Lyso PI, 18:0 Lyso PI, 16:0 PE, 14:0 PC DMPC, 16:0 ceramide, 18:0 ceramide in negative ion mode, and 17:0 PE, 19:0 PC, 14:0 Lyso PC, 20:0 Lyso PC, 18:0 PC DSPC, 18:0–22:6 PC in positive ion mode were significantly altered only in ICM as compared to IHD and control.

**Conclusion:**

Using non-targeted lipidomics profiling, we have successfully identified a group of circulating lipids that were significantly altered in IHD and ICM. The lipids metabolic signatures shed light on potential new biomarkers and therapeutics for preventing and treating ICM.

## Background

Ischemic heart disease (IHD), also referred to as coronary heart disease, is associated with inadequate supply of blood to the myocardium. Patients are described as stable when symptoms are manageable with either medical or revascularization therapy [1]. Chronic coronary ischemia could cause significantly impaired left ventricular function, leading to ischemic cardiomyopathy (ICM) [2, 3]. It has been proven that IHD are related to profound metabolic alterations, as heart suffers from intermittent ischemia and hypoxia [4]. It is also worth mentioning that metabolism disorders, such as dyslipidemia, diabetes mellitus and increased alcohol intake are associated with an increased incidence of IHD [5]. Yet few studies have utilized metabolomics approach to describe metabolic profile and perturbance in IHD, especially during different levels of cardiac function lesions.

Metabolomics has been emerging as a powerful tool for defining changes in both global and cardiac-specific metabolism that occur across a spectrum of cardiovascular disease [6]. As an important branch of metabolomics, lipidomics describes the spatial and temporal alterations in the content and composition of different lipid molecules [7]. Although metabolic disturbances have been well established in IHD, few studies have discussed metabolic alterations based on lipidomics.

It has been proven that cardiomyopathy is associated with profound changes in cardiac metabolism. Pathological progression of ICM results in cardiac structural remodeling, leading to an increased reliance on glucose metabolism and decrease in fatty acid oxidation [8, 9]. Moreover, during the transition from cardiac hypertrophy to failure, mitochondrial number and function progressively decline, leading to an overall decrease in the oxidative metabolites [10, 11] Metabolic disturbances have been previously in-depth investigated in heart failure. Yet few studies have analyzed metabolic profiles in ischemic cardiomyopathy, especially during the progression from IHD to ICM. Thus, elucidating lipids metabolic profile alteration and identifying novel circulatory markers are of critical importance in the treatment and evaluation of IHD and ICM.

In this study, by utilizing lipidomics approach, we aim to characterize and compare the serum lipids metabolic profile in IHD and ICM patients. We have found significant alterations in a number of lipids levels, which are more prominent in ICM patients. Altered serum lipids exhibit diagnostic value for ICM and are closely correlated to clinical factors. Applied together, lipids profiling could be applied to identify patients in disease progression from IHD to ICM, and thus potentially add to our diagnostic armamentarium.

## Materials and methods

### Study design and population

Consecutive consenting patients less than 70 yrs. admitted to the cardiology department of the First Affiliated Hospital of Xi’an Jiaotong University for chest pain who subsequently underwent coronary angiography from February 2018 to August 2018 were screened. IHD and ICM were defined according to the universal definition criteria by the American Cardiology College respectively [12]. The IHD was defined as: 1) Preserved myocardial function characterized by EF > 50%; 2) Impaired blood flow with more than 50% stenosis of coronary arteries; 3) Angina that occurs with exercise and is predictable, usually promptly relieved by rest or nitroglycerin. ICM were diagnosed upon: 1) More than 50% stenosis of coronary arteries confirmed by angiography; 2) Impaired myocardial function characterized by EF<40%. The exclusion criteria were: 1) Acute decompensated heart failure; 2) Acute myocardial infarction; 3) Unstable hemodynamics; 4) Hepatic, nephritic, hematological or autoimmune disorders; 5) Severe noncardiac disease with expected survival of less than 1 year; 6) Cachexia; 7) Patients over the age of 80 years; 8) Unwillingness to participate.

Among more than 5000 patients screened, 642 patients met the inclusion and exclusion criteria, including 501 IHD and 141 ICM patients. Written informed consent was obtained from 364 patients. Of these, 25 IHD and 25 ICM patients were selected for serum lipidomics assessments. Twenty-five volunteers with coronary atherosclerosis less than 50% by angiography were randomly selected as control. Demographic and biochemical information was obtained as previously described [13–15].

### Serum sample preparation

Serum samples were collected from IHD, ICM and control patients after coronary angiography. Venous blood was withdrawn the next morning after overnight fast and immediately centrifuged at 3000 rpm for 10 min at 4 °C. Serum was separated and stored at − 80 °C and aliquots were thawed for further processing as previously described [13, 15].

### Determination of serum lipids profile

Before analysis, serum samples were collected and thawed. 10 μl serum was mixed with 10 μl 0.9%Nacl, 10 μl internal control solution (isopropanol-acetonitrile with 1 μg/mL 19:0–19:0 PC, 17:0–17:0 PE, 12:0 SM and 19:0 Lyso PC respectively). The mixture solution was vortexed for 20s and stabled for 30 min in 4 °C. The mixture was centrifuged at 7800 g/min for 3 min. Supernatant was removed and leftover was transferred to 2 tubes, dried with nitrogen and kept in − 20 °C for positive and negative measurements afterwards. To perform the serum lipid analysis, the dried sample was resuspended in 20 μl isopropanol-acetonitrile and vortexed for 60s. Lipid profling was performed by Eksigent LC100 and AB SCIEX Triple TOF 5600+ with mass spectrometer Waters XBridge Peptide BEH C18 3.5 μm, 2.1 × 100 mm in positive ion mode and negative ion mode. The lipidomics data was processed by the software PeakView1.2 for qualitative analysis and MultiQuant2.1 for quantitative analysis.

### Statistical analysis

Data were normalized using MetaboAnalyst before analyses as previously described [16–25] (Supplementary Figures S1 and S2). Lipid values below the lower limit of detection were excluded from these analyses. Partial Least Squares Discriminant Analysis (PLS-DA) models were employed to reduce a large number of correlated metabolites to a smaller number of uncorrelated factors. Individual lipids levels among three groups were compared using one-way ANOVA. Data were presented as mean ± SE. *P*-values < 0.05 were considered as significant * < 0.05, ** < 0.01, and *** < 0.001. Receiver operating characteristics (ROC) was used and areas under the ROC curve (AUC) were calculated to explore the discriminative capability of different lipids to identify ICM. Pearson analysis was performed to compare the interrelation between lipids and biochemical indicators and heat map was created using R studio.

## Results

### Baseline characteristics

A total of 75 patients were enrolled in the study, including 25 IHD, ICM and 25 control patients. Table 1 described the demographic and biochemical characteristics of the enrolled patients. The mean age was 59.89 ± 16.81 for ICM, 60.48 ± 8.94 for IHD and 52.71 ± 16.09 for control participants. The mean left ventricular ejection fraction (LVEF) was 34.53 ± 7.57 for ICM, 64.14 ± 9.27 for IHD and 68.30 ± 5.46 for control patients. No significant differences at baseline were seen in age, heart rate (HR), aspartate transaminase (AST), alanine aminotransferase (ALT), creatinine (CRE), total cholesterol (TC), triglycerides (TG) high and low density lipoprotein cholesterol (HDL-C, LDL-C).

**Table 1:** Basic charectersticsbased in different patient groups

	ICM	IHD	Control	*P* value
**Female(%)**	32.00%	52.00%	44.00%	
**Age(y)**	59.9 ± 16.8	60.5 ± 8.9	52.7 ± 16.1	ns
**HR (bpm)**	74.11 ± 23.61	68.57 ± 10.92	74.96 ± 12.65	ns
**sBP (mmHg)**	118.98 ± 31.49	129.96 ± 21.83	121.46 ± 12.79	< 0.05
**dBP (mmHg)**	67.71 ± 19.23	71.26 ± 9.67	71.83 ± 12.11	< 0.01
**EF(%)**	44.53 ± 18.57	64.14 ± 9.27	68.30 ± 5.46	< 0.001
**AST(U/L)**	20.64 ± 6.92	21.79 ± 7.22	21.03 ± 6.15	ns
**ALT(U/L)**	26.81 ± 13.42	25.18 ± 16.57	19.02 ± 12.82	ns
**CRE (mg/dL)**	68.58 ± 26.33	65.17 ± 12.38	58.92 ± 12.98	< 0.05
**CHOL (mmol/L)**	3.65 ± 1.44	3.87 ± 0.91	4.31 ± 1.14	ns
**TG (mmol/L)**	1.54 ± 0.72	1.80 ± 1.14	1.42 ± 0.76	ns
**HDLC (mmol/L)**	0.84 ± 0.25	0.95 ± 0.22	1.19 ± 0.35	< 0.001
**LDLC (mmol/L)**	2.25 ± 1.17	2.27 ± 0.77	2.50 ± 0.87	ns
**PROBNP (ng/mL)**	9190.28 ± 7955.63	415.15 ± 722.46	124.64 ± 115.36	< 0.001
**In hospital treatment**				
**Aspirin(%)**	100.0%	100.0%	52.0%	
**clopidogrel(%)**	100.0%	60.0%	52.0%	
**Anticoagulants(%)**	20.0%	4.0%	0.0%	
**AECI(%)**	68.0%	52.0%	12.0%	
**ARB(%)**	20.0%	28.0%	8.0%	
**β-blocker(%)**	92.0%	92.0%	8.0%	
**Statin(%)**	100.0%	100.0%	100.0%	
**nitrate(%)**	60.0%	52.0%	4.0%	
**CCB(%)**	12.0%	20.0%	20.0%	
**Spironolactone(%)**	80.0%	0.0%	0.0%	
**Stent afer angiography(%)**	76.0%	72.0%	0.0%	

*Abbreviations*: *HR* Heart Rate, *BP* blood pressure, *EF* Ejection Fraction, *AST* Aspartate transaminase, *ALT* Alanine aminotransferase, *CRE* Creatinine, *CHOL* Total Cholesterol, *TG* Triglycerides, *HDL* High Density Lipoprotein Cholesterol, *LDL* Low Density Lipoprotein Cholesterol, *proBNP* pro Brain Natriuretic Peptide

### Non-targeted lipidomics analysis

**Fig. 1:**
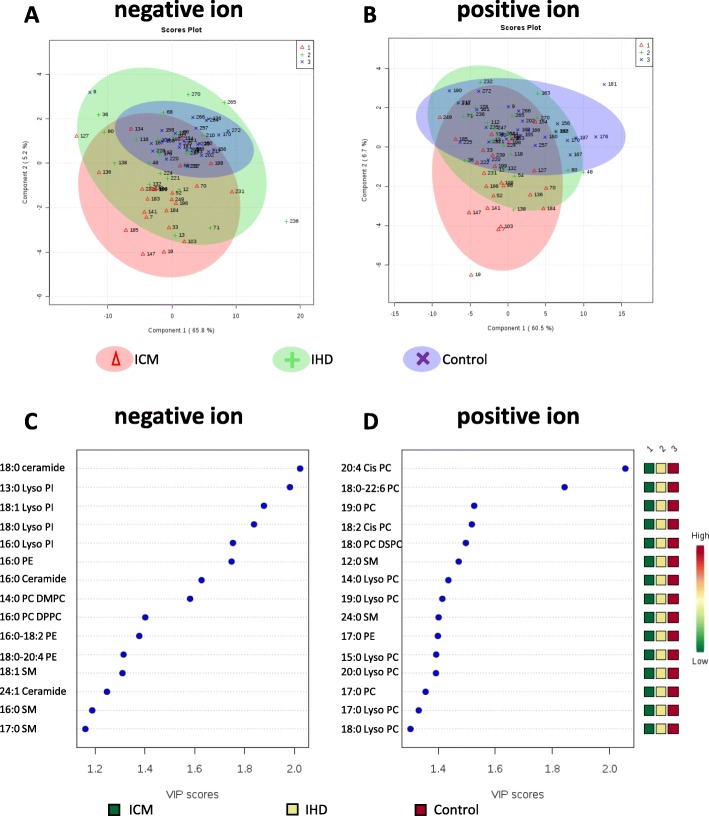
PLS-DA score plots of IHD, ICM and control patients based on lipidomics data. **a** 2D-projection plots of lipids from PLS-DA for the of IHD, ICM and control patients in negative ion mode. **b** 2D-projection plots of lipids from PLS-DA for the of IHD, ICM and control patients in positive ion mode. The discrimination plane between 3 groups was obtained by linear discrimination analysis (LDA). **c** Variable importance in projection (VIP) of lipids rested in negative ion mode. **d** VIP of lipids rested in positive ion mode

Firstly, we employed PLS-DA for profile visualization and differentiation among the multiple groups. The initial overview of global metabolic profiles as revealed by PLS-DA scores plot indicated that lipids among IHD, ICM and control group were generally correlated but to some extent separable, which is more prominent for lipids in positive ion mode (Fig. 1). The heatmap overview for the serum lipids levels were shown in supplemental Figures S1 and S2. The variable importance in projection (VIP) for lipids in negative and positive ion mode were shown in Fig. 1c and Fig. 1d, which indicated the importance in projection of listed lipids.

**Fig. 2:**
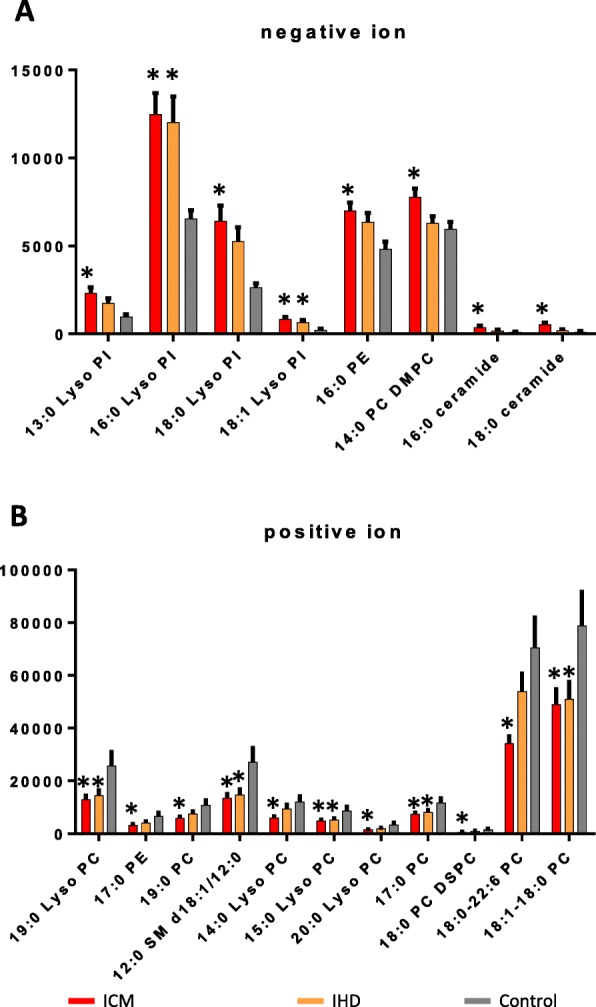
Relative levels of significantly altered metabolites based on lipidomics. **a**. Relative levels of significantly altered lipids in IHD, ICM and control patients in negative ion mode. **b**. Relative levels of significantly altered lipids in IHD, ICM and control patients in positive ion mode. Data were analyzed using one-way ANOVA. Mean ± s.e.m. * *p* < 0.05 as compared to control

**Fig. 3:**
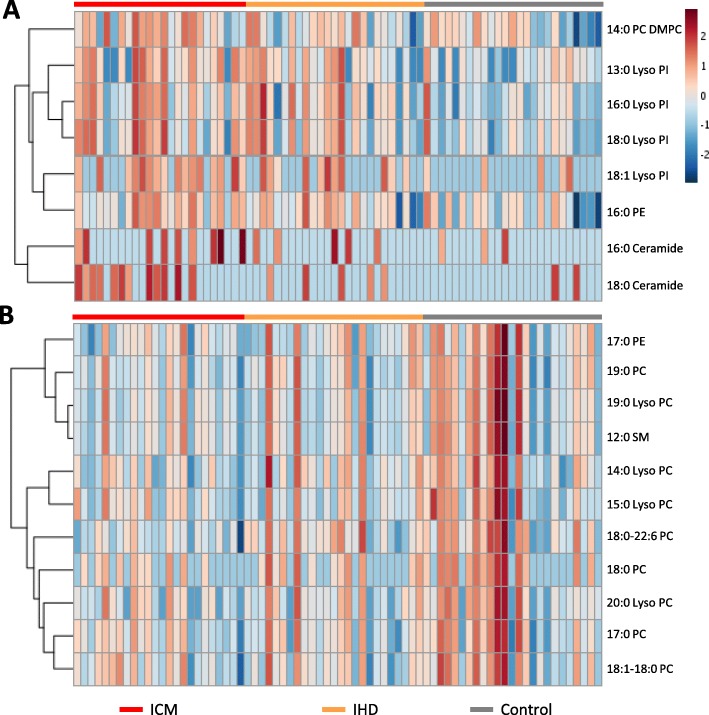
Heatmaps of significantly altered metabolites based on lipidomics. **a** Heatmap of the significantly altered lipids in IHD, ICM and control patients in negative ion mode. **b** Heatmap of the significantly altered lipids in IHD, ICM and control patients in positive ion mode. The colors in the heat map indicated the log transformed values of each metabolites

To identify the individual lipids levels, we compared the lipids levels in negative and positive ion modes among IHD, ICM and control patients using one-way ANOVA (Fig. 2). A total of 63 lipids were detected in negative ion mode (Supplementary Table S1). Among them, 16:0 lysophosphatidylinositol (Lyso PI) and 18:1 Lyso PI were significantly altered both in IHD and ICM; 13:0 Lyso PI, 18:0 Lyso PI, 16:0 phosphatidylethanolamine (PE), 14:0 phosphatidylcholine dimyristoylphosphatidylcholine (PC DMPC), 16:0 ceramide and 18:0 ceramide were significantly altered only in ICM as compared to IHD and control (Fig. 2a). In positive ion mode, a total of 62 lipids were detected (Supplementary Table S1). Among them, 19:0 lysophosphatidylcholine (Lyso PC), 12:0 sphngomyelin (SM) d18:1/12:0, 15:0 Lyso PC, 17:0 phosphatidylcholine (PC) and 18:1–18:0 PC were significantly altered both in IHD and ICM; 17:0 PE, 19:0 PC, 14:0 Lyso PC, 20:0 Lyso PC, 18:0 phosphatidylcholine (PC DSPC) and 18:0–22:6 PC were significantly altered only in ICM as compared to IHD and control (Fig. 2b). A heatmap of the individual levels of significantly altered serum lipids (*p* < 0.05) is shown in Fig. 3.

### Receiver operating characteristics (ROC) analysis

**Fig. 4:**
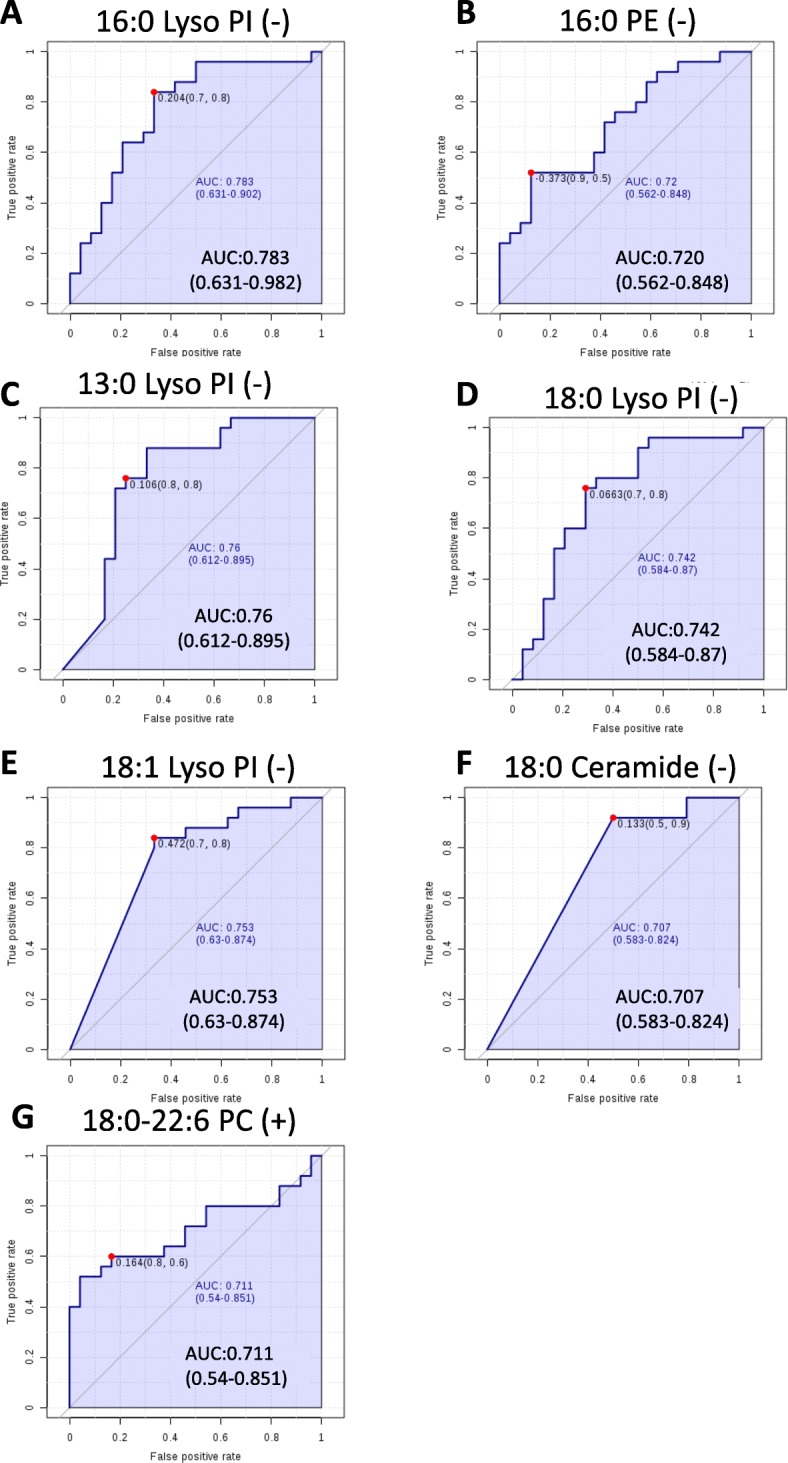
Receiver operating characteristics (ROC) analysis of different lipids to identify ICM patients. **a**-**g** Receiver operating characteristic (ROC) curve analysis for 6 lipids detected in negative ion mode(**a**-**f**) and 1 lipid detected in positive ion mode (**g**). Area under the ROC curve and confidence interval for each lipids were shown in each figure

Since non-targeted lipidomics analysis had identified significant alterations between ICM and control, we further performed ROC analysis for discovery and identification of potential lipid biomarkers. Among 19 significantly altered lipids tested in negative and positive ion mode, 16:0 Lyso PI, 16:0 PE, 13:0 Lyso PI, 18:1 Lyso PI, 18:0 ceramdie and 18:0 Lyso PI detected in negative ion mode (Fig. 4a-f) and 18:0–22:6 PC (Fig. 4g) in positive ion mode showed diagnostic value for ICM with the area under the ROC curve (AUR) all above 0.7 and *P* value< 0.05.

### Interrelation between lipids and clinical characters

**Fig. 5:**
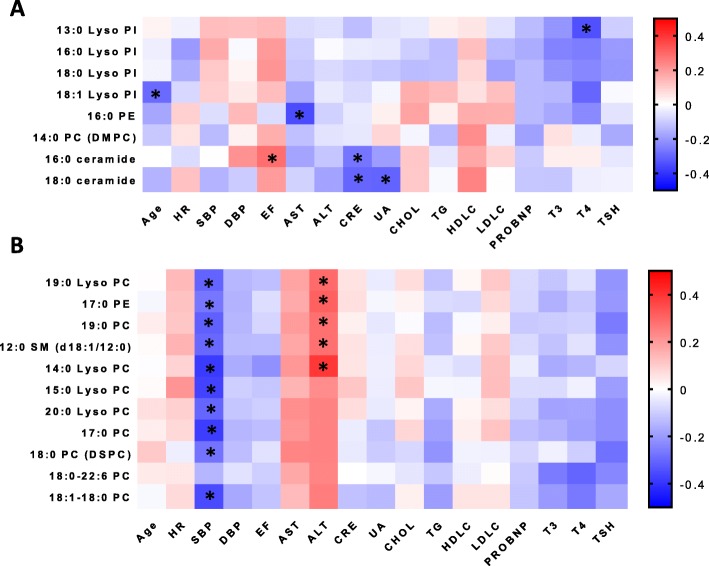
Correlation of lipids to clinical factors in cohort. **a** Correlations between age, HR, blood pressure, EF, hepatic function, renal function, serum lipid levels, thyroid function and lipids profile in negative ion mode; **b** Correlations between age, HR, blood pressure, EF, hepatic function, renal function, serum lipid levels, thyroid function and lipids profile in positive ion mode; The colors within each crossover represented the correlation efficiency between the respective lipids and clinical factors of the Pearson’s correlation. Blue color indicated decreased correlation and red color indicated enhanced correlation. * *p* < 0.05

At last, we explored the interrelationship between the significantly altered lipids and the clinical phenotype. Figure 5 showed the heatmap of the Pearson’s correlation coefficients between age, HR, blood pressure, EF, hepatic function, renal function, serum lipid levels, thyroid function and lipids profile in negative ion mode (Fig. 5a) and positive ion mode (Fig. 5b). Red squares indicated the highest positive coefficient of 1 and blue squares indicated the lowest negative coefficient of − 1. It was noteworthy that the 16:0 ceramide and 18:0 ceramide were both significantly and negatively correlated to renal function as indicated by serum creatinine. It was also quite interesting to notify the prominent negative correlation between systolic blood pressure and nearly all altered lipids in positive ion mode except for 18:0–22:6 PC.

## Discussion

In this study, metabolic profile and the network of serum lipids are analyzed in IHD and ICM patients. Lipids metabolic perturbance is observed in both IHD and ICM based on the following results: 1) A number of lipids are altered in IHD and ICH; 2) Lipids metabolic alterations are more significant in ICM and most of altered serum lipids also show significant diagnostic value for ICM; 3) Serum lipids profile exhibit interrelation to clinical features among ICM patients.

Consistent to previous publications, our present lipidomics analysis further confirms metabolic alterations as myocardial undergoes ischemia. Recent technological advances have enabled integration of multiple layers analysis from genome, epigenome, transcriptome, proteome, metabolome to even the microbiome [5]. Previous metabolic approaches have also identified that differences in small-molecule metabolites may provide biomarkers for coronary artery disease progression [4, 26–28]. In correlation to previous investigation, we have further confirmed the metabolic perturbance of PE and PC during myocardial ischemia. However, our study indicate specific altered subunits of PE and PC, which are helpful in further mechanism investigation.

It is also interesting to notify that, based on lipidomics approach in positive ion mode, significantly altered lipids are closely negatively related to systolic blood pressure, although not to cardiac ejection fraction. It is well known that sBP is regulated by cardiac contraction function, cardiac output, and aortic resilience. As cardiac ejection fraction show no correlation to lipids both in negative and positive ion mode, and most of correlated lipids ascribe to phosphocholine, we suppose that decreased aorta resilience, especially during ICM, is associated with lower phosphocholine. However, the hypothesis from this cross-sectional observational study warrants further validation.

The novelty of the current study is that we have provided new lipidomic alteration evidence as heart undergoes transition from IHD to ICM. The term ICM describes significantly impaired left ventricular function resulting from IHD. Previous analyses of conventional metabolism and circulating metabolites have confirmed metabolic disturbances as heart undergoes structural and functional change from IHD to ICM [29, 30]. Cardiac pathological structural remodeling has resulted in reprogramming of cardiac metabolic pathways. The metabolic consequences of ICM have been examined in a wide variety of experimental animal models. Present analysis has indicated that serum lipids have undergone profound alterations as myocardial ischemia progressing to myopathy. The altered lipids could serve as potential biomarkers for prognosis of ICM. Identification for deeper molecular mechanism could be helpful for understanding pathophysiologic alterations.

Recent advances in metabolic profiling technologies have enhanced the feasibility of high-throughput patient screening for the diagnosis of disease state [31]. However, applying global metabolomics approach to explore cardiovascular disease is still lacking, and prior studies have generally assayed relatively limited subsets of metabolites in focused approaches. In the present study, we utilized non-targeted metabolomics analysis to investigate lipidomic alterations in patients with different levels of myocardial ischemia. Circulating lipids are found to participate as regulatory signals and could be potential biomarkers for ICM. The large-scale metabolic profiling approaches have been recognized as a more comprehensive survey to better inform underlying biological processes and identify potential biomarkers for disease progression in the present analysis.

Our study has several limitations. Although we have studied 3 major groups along the myocardial spectrum, the cohort size for each group is relatively limited, and the selection and observation bias could not be easily excluded. In addition, serum lipids alterations are based on non-targeted metabolic approach and warrant further confirmation by targeted metabolomics measurement. Moreover, the present investigation is agnostic to tissue source of circulating metabolites. Thus, molecular and biochemical confirmation are also required to further explain the exact metabolic pathway alterations. Since this is a clinical observational study, we could not exclude the confounding factor of the drugs, procedures, etc. Large-scale and long-time follow-up studies are also necessary to validate the diagnostic function of lipids for disease phenotype, and to evaluated the lipidomic markers between patients with differences in coronary angiography.

## Conclusion

In this study, we have demonstrated the application of lipidomics platform to better understand pathogenic progression from IHD to ICM. Lipidomics profile is more significantly altered as myocardial ischemia progresses to ischemic cardiomyopathy. The lipids metabolic signatures also provide novel biomarkers for preventing and diagnosing IHD and ICM.

## Supplementary information

**Additional file 1.**

## Data Availability

The datasets used and/or analysed during the current study are available from the corresponding author on reasonable request.

## Abbreviations

IHD: Ischemic heart disease
ICM: Ischemic cardiomyopathy
LVEF: Left ventricular ejection fraction
HR: Heart rate
AST: Aspartate transaminase
ALT: Alanine aminotransferase
CRE: Creatinine
UA: Urea acid
CHOL: Total cholesterol
TG: Triglycerides
HDL: High density lipoprotein cholesterol
LDL: Low density lipoprotein cholesterol
T3: Triiodothyronine
FT4: Free thyroxine
TSH: Thyroid stimulating hormone
PLS-DA: Partial least squares discriminant analysis
VIP: Variable importance in projection
ROC: Receiver operating characteristics
