# Supplementary material for: Serum lipids profiling perturbances in patients with ischemic heart disease and ischemic cardiomyopathy
Source: Lipids Health Dis. 2020 May 9;19:89. doi: 10.1186/s12944-020-01269-9 (PMC7210665; doi:10.1186/s12944-020-01269-9)
Supplement: Supplementary file 1 — Additional file 1. [file 12944_2020_1269_MOESM1_ESM.pptx]

## Slide 1
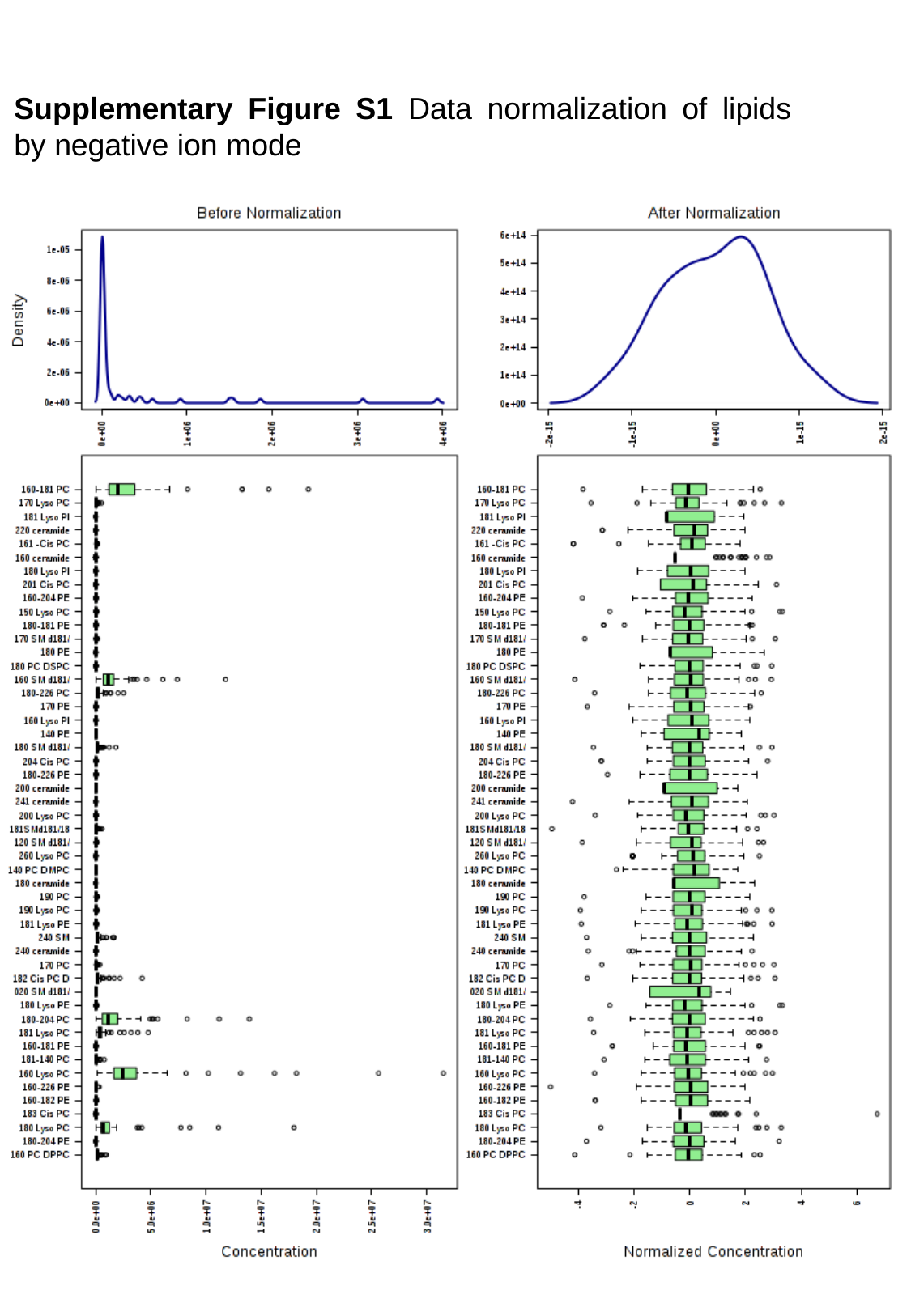

Supplementary Figure S1 Data normalization of lipids by negative ion mode

## Slide 2
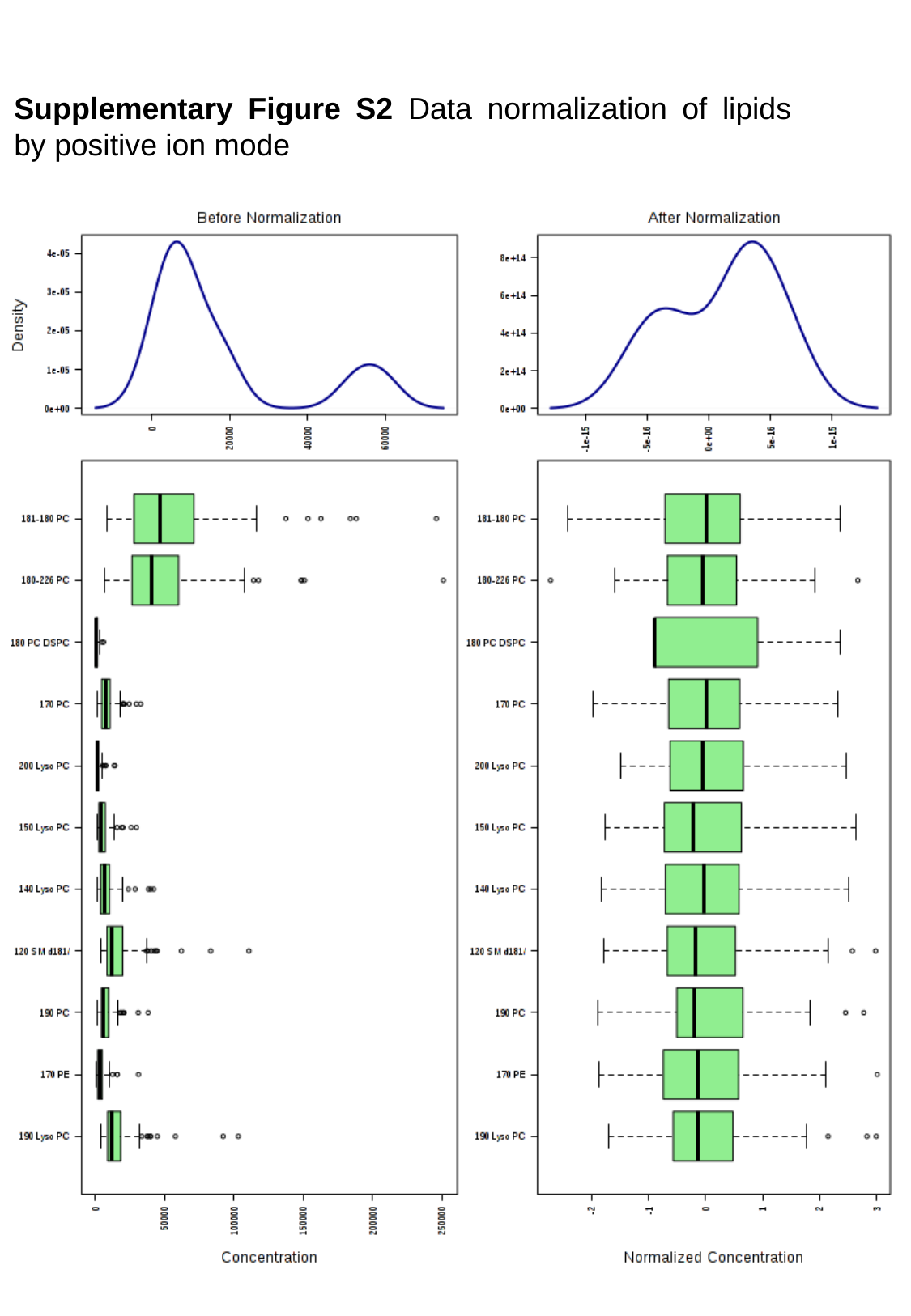

Supplementary Figure S2 Data normalization of lipids by positive ion mode

## Slide 3
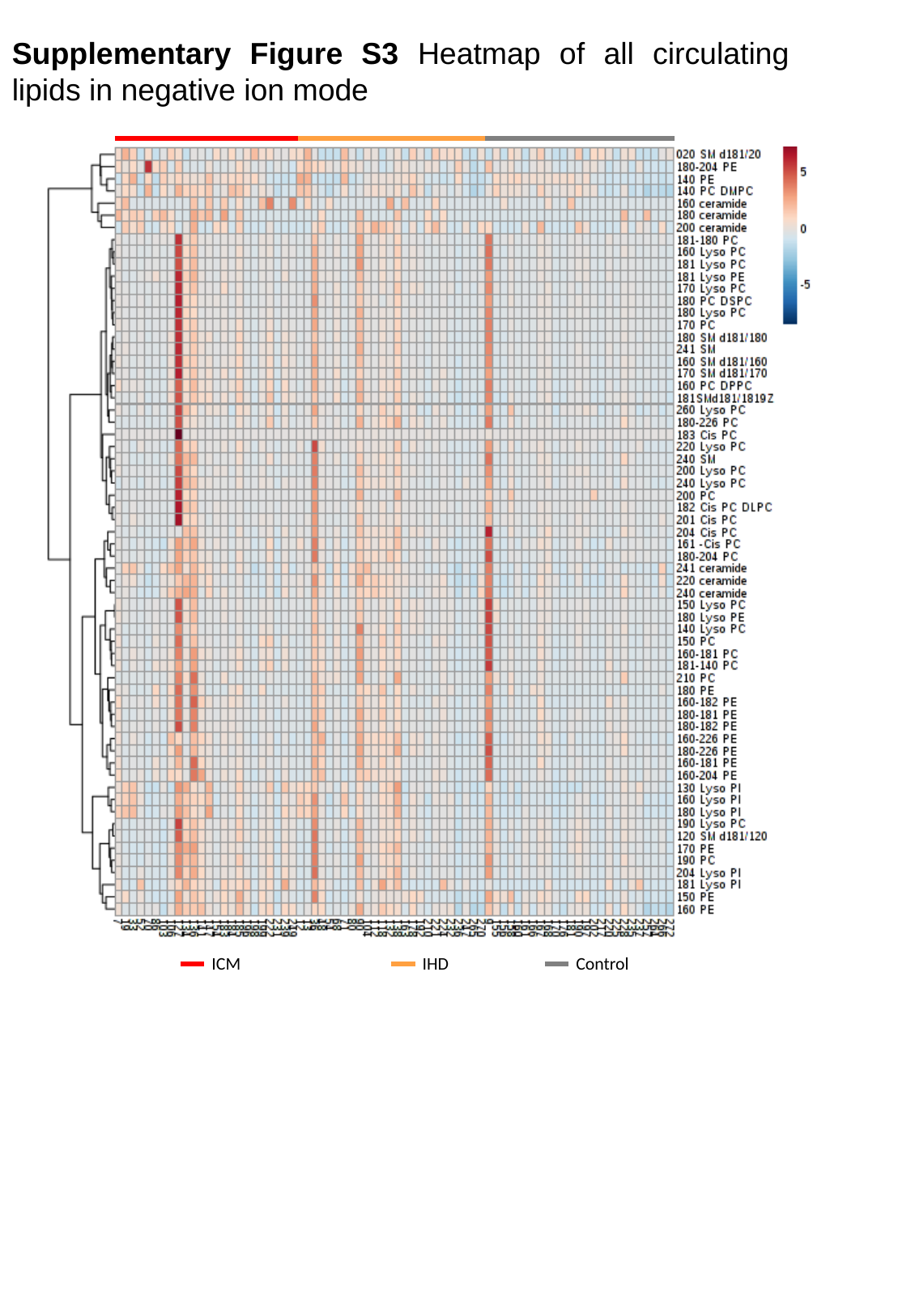

Supplementary Figure S3 Heatmap of all circulating lipids in negative ion mode
ICM
IHD
Control

## Slide 4
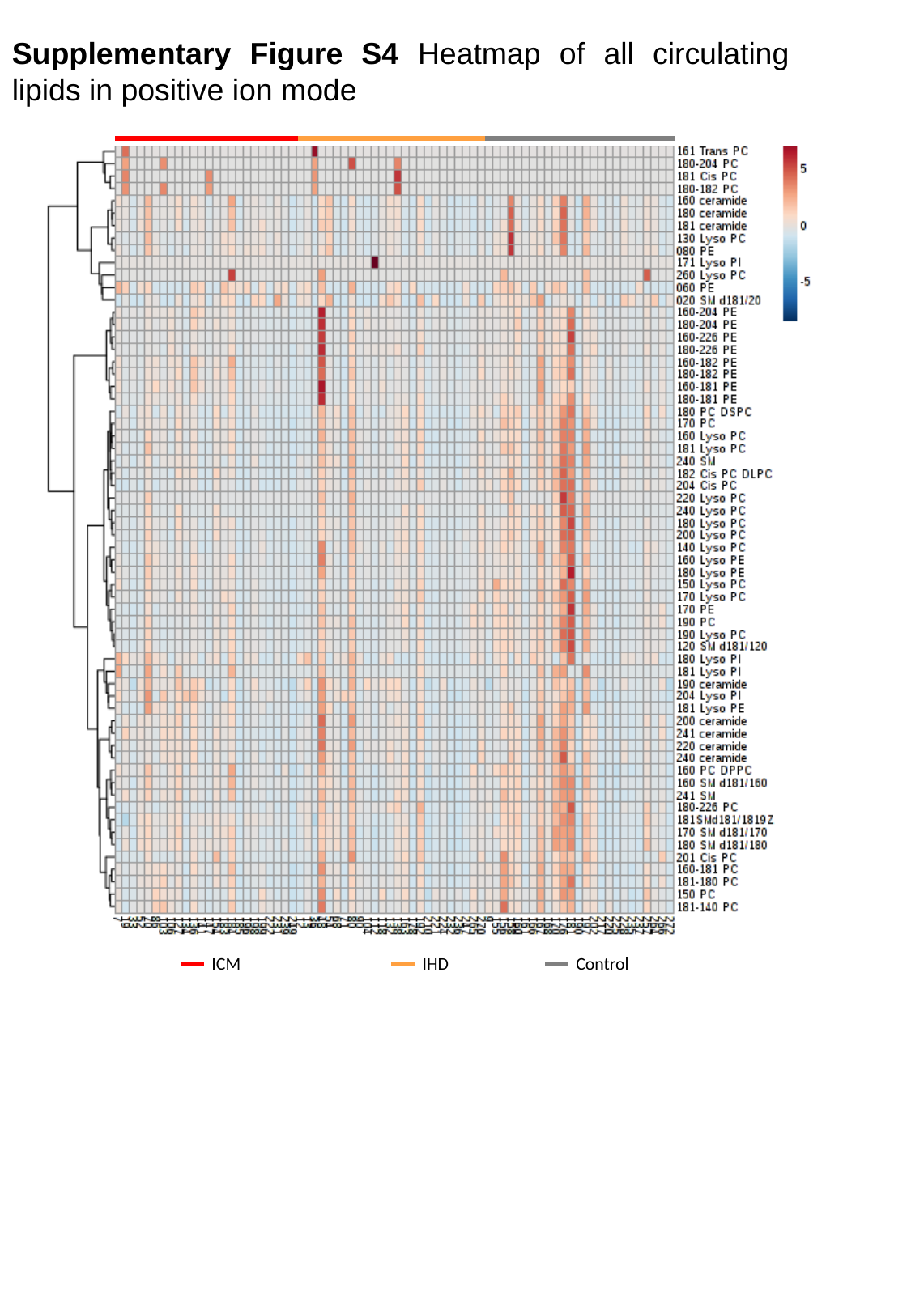

Supplementary Figure S4 Heatmap of all circulating lipids in positive ion mode
ICM
IHD
Control

## Slide 5
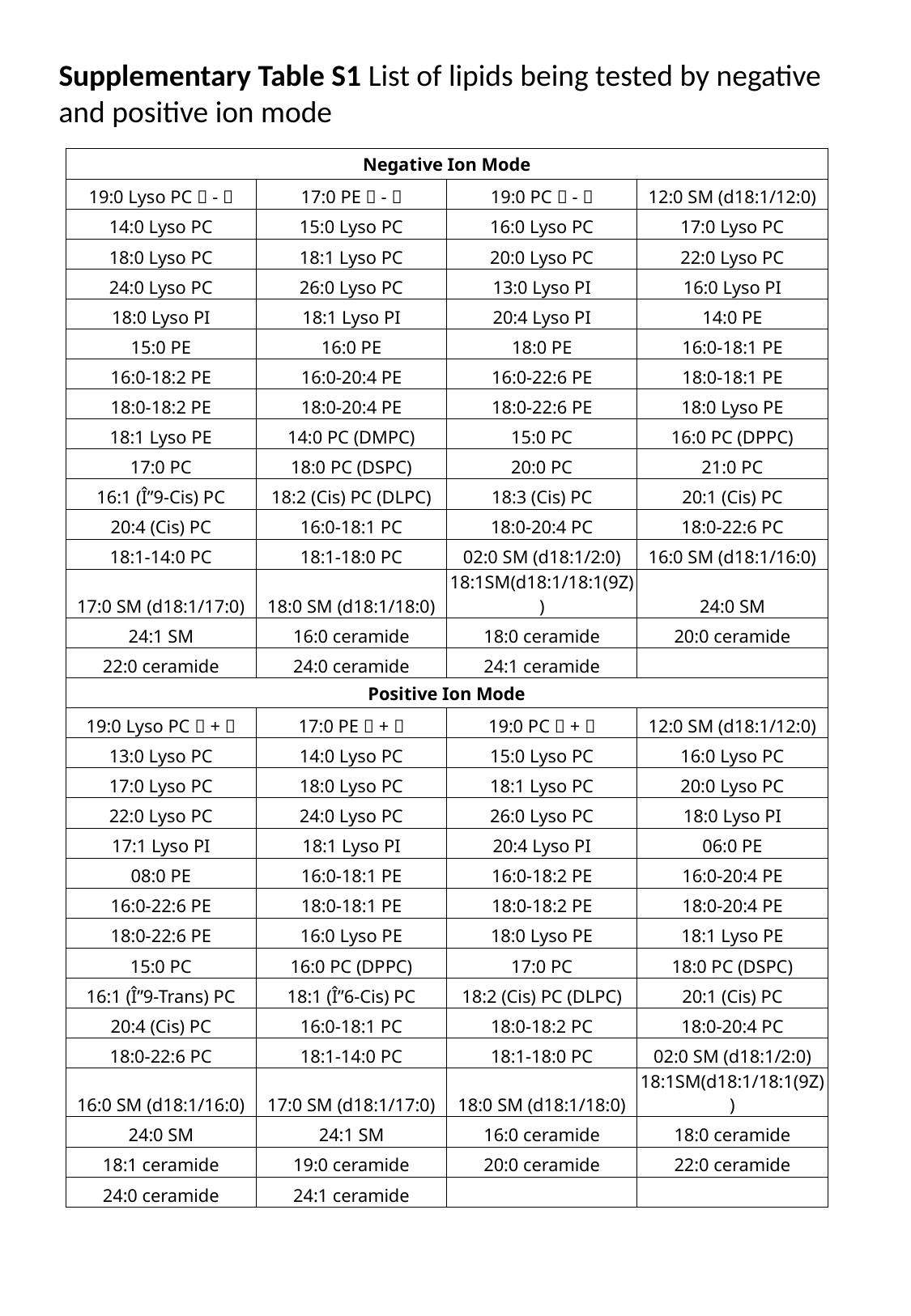

Supplementary Table S1 List of lipids being tested by negative and positive ion mode
| Negative Ion Mode | | | |
| --- | --- | --- | --- |
| 19:0 Lyso PC（-） | 17:0 PE（-） | 19:0 PC（-） | 12:0 SM (d18:1/12:0) |
| 14:0 Lyso PC | 15:0 Lyso PC | 16:0 Lyso PC | 17:0 Lyso PC |
| 18:0 Lyso PC | 18:1 Lyso PC | 20:0 Lyso PC | 22:0 Lyso PC |
| 24:0 Lyso PC | 26:0 Lyso PC | 13:0 Lyso PI | 16:0 Lyso PI |
| 18:0 Lyso PI | 18:1 Lyso PI | 20:4 Lyso PI | 14:0 PE |
| 15:0 PE | 16:0 PE | 18:0 PE | 16:0-18:1 PE |
| 16:0-18:2 PE | 16:0-20:4 PE | 16:0-22:6 PE | 18:0-18:1 PE |
| 18:0-18:2 PE | 18:0-20:4 PE | 18:0-22:6 PE | 18:0 Lyso PE |
| 18:1 Lyso PE | 14:0 PC (DMPC) | 15:0 PC | 16:0 PC (DPPC) |
| 17:0 PC | 18:0 PC (DSPC) | 20:0 PC | 21:0 PC |
| 16:1 (Î”9-Cis) PC | 18:2 (Cis) PC (DLPC) | 18:3 (Cis) PC | 20:1 (Cis) PC |
| 20:4 (Cis) PC | 16:0-18:1 PC | 18:0-20:4 PC | 18:0-22:6 PC |
| 18:1-14:0 PC | 18:1-18:0 PC | 02:0 SM (d18:1/2:0) | 16:0 SM (d18:1/16:0) |
| 17:0 SM (d18:1/17:0) | 18:0 SM (d18:1/18:0) | 18:1SM(d18:1/18:1(9Z)) | 24:0 SM |
| 24:1 SM | 16:0 ceramide | 18:0 ceramide | 20:0 ceramide |
| 22:0 ceramide | 24:0 ceramide | 24:1 ceramide | |
| Positive Ion Mode | | | |
| 19:0 Lyso PC（+） | 17:0 PE（+） | 19:0 PC（+） | 12:0 SM (d18:1/12:0) |
| 13:0 Lyso PC | 14:0 Lyso PC | 15:0 Lyso PC | 16:0 Lyso PC |
| 17:0 Lyso PC | 18:0 Lyso PC | 18:1 Lyso PC | 20:0 Lyso PC |
| 22:0 Lyso PC | 24:0 Lyso PC | 26:0 Lyso PC | 18:0 Lyso PI |
| 17:1 Lyso PI | 18:1 Lyso PI | 20:4 Lyso PI | 06:0 PE |
| 08:0 PE | 16:0-18:1 PE | 16:0-18:2 PE | 16:0-20:4 PE |
| 16:0-22:6 PE | 18:0-18:1 PE | 18:0-18:2 PE | 18:0-20:4 PE |
| 18:0-22:6 PE | 16:0 Lyso PE | 18:0 Lyso PE | 18:1 Lyso PE |
| 15:0 PC | 16:0 PC (DPPC) | 17:0 PC | 18:0 PC (DSPC) |
| 16:1 (Î”9-Trans) PC | 18:1 (Î”6-Cis) PC | 18:2 (Cis) PC (DLPC) | 20:1 (Cis) PC |
| 20:4 (Cis) PC | 16:0-18:1 PC | 18:0-18:2 PC | 18:0-20:4 PC |
| 18:0-22:6 PC | 18:1-14:0 PC | 18:1-18:0 PC | 02:0 SM (d18:1/2:0) |
| 16:0 SM (d18:1/16:0) | 17:0 SM (d18:1/17:0) | 18:0 SM (d18:1/18:0) | 18:1SM(d18:1/18:1(9Z)) |
| 24:0 SM | 24:1 SM | 16:0 ceramide | 18:0 ceramide |
| 18:1 ceramide | 19:0 ceramide | 20:0 ceramide | 22:0 ceramide |
| 24:0 ceramide | 24:1 ceramide | | |
